# Self-regulation of time: The importance of time estimation accuracy

**DOI:** 10.3389/fpsyg.2022.925812

**Published:** 2022-10-24

**Authors:** Anna C. Brady, Christopher A. Wolters, Shirley L. Yu

**Affiliations:** ^1^Department of Curriculum, Foundations, and Reading, College of Education, Georgia Southern University, Statesboro, GA, United States; ^2^Dennis Learning Center, Department of Educational Studies, College of Education and Human Ecology, The Ohio State University, Columbus, OH, United States; ^3^Department of Educational Studies, College of Education and Human Ecology, The Ohio State University, Columbus, OH, United States

**Keywords:** self-regulated learning, time management, time estimation, planning fallacy, college students

## Abstract

Time management is one central aspect of students’ self-regulated learning. In addition, biased time estimation seems to be central to students’ self-regulation of their time. In this study, we explored college students’ time estimation bias. In addition, we were interested in whether the activation of task beliefs influenced students’ time estimation bias and how specific beliefs about task difficulty influence time estimation bias. Findings suggested that students tended to demonstrate bias in their estimations of the time their academic tasks would take. Additionally, the activation of task beliefs did not influence students’ time estimation accuracy. Finally, both prior task difficulty and anticipated difficulty influenced students’ time estimation bias. These findings highlight the complexity of students’ time estimation bias and point to the opportunities for future directions.

## Introduction

Life as a college student includes a wide range of activities and responsibilities. The effective management of tasks, particularly academic tasks, is essential for college students’ success (Zimmerman et al., 1994). One way that students manage their academic tasks and other responsibilities is through time management strategies. Researchers have suggested that the process of time management is important for academic success (Claessens et al., 2007; Kitsantas et al., 2008; Aeon and Aguinis, 2017). Due to the clear link between time management and academic performance, researchers have turned their attention toward understanding the specific processes involved in time management (Wolters and Brady, 2021). The purpose of the present study was to investigate one process related to time management: time estimation bias. Specifically, the present study investigated college students’ tendency to accurately estimate the amount of time it would take them to complete academic tasks. Using a quasi-experimental approach, we also examined whether activating task beliefs influences students’ time estimation bias. In addition, we examined the impact of perceived difficulty on students’ time estimation.

### Time estimation

Time estimation is the process of approximating the amount of time a task will take prior to beginning the task (Buehler et al., 1994). To appropriately plan for the completion of academic tasks, a student must be able to realistically identify how much time a task will take (Wolters and Brady, 2021). The majority of research focused on time estimation has investigated time estimation accuracy and bias (as reviewed by Halkjelsvik and Jørgensen, 2012; Buehler and Griffin, 2015). Although research tends to suggest that people are biased in their time estimation predictions, there is not agreement on whether individuals tend to overestimate or underestimate their time on tasks (Halkjelsvik and Jørgensen, 2012). Some researchers have argued that people tend to underestimate how long tasks will take (Buehler et al., 1997; Roy et al., 2005; Boltz and Yum, 2010; Buehler and Griffin, 2015), while others have noted that the body of work focused on time estimation varies in whether people over or underestimate their time on tasks (Halkjelsvik and Jørgensen, 2012). Early research on this topic termed individuals’ biased estimations as the “planning fallacy” (Kahneman and Tversky, 1977).

People’s time estimation has been commonly investigated using two different research designs: naturalistic studies focused on long-term tasks and experimental studies focused on short-term tasks. In addition, researchers have distinguished between performance time predictions (i.e., estimating how many minutes a task will take) and completion time predictions (i.e., estimating the particular date or time when a task will be completed; Halkjelsvik and Jørgensen, 2012). Naturalistic studies focus on authentic academic and non-academic tasks that individuals plan to complete by asking individuals to estimate the date by which they plan to complete a particular task. Typically, these studies focus on multifaceted and important longer-term tasks that individuals plan to complete over multiple episodes of engagement (Buehler et al., 1994; Weick and Guinote, 2010). In addition, in these studies researchers do not typically examine the number of minutes tasks will take; rather, they investigate inconsistencies in the date by which individuals expect to complete their task. For example, Buehler et al. (1994) asked undergraduate students who were completing an honors thesis to estimate the date on which they planned to submit their thesis. They found that students tended to demonstrate inaccuracy in the date they planned to submit their honors thesis by; they estimated submitting it earlier than the date they actually submitted their thesis (Buehler et al., 1994). A different study conducted by Weick and Guinote (2010) asked 20 undergraduate students to estimate their completion of a final course project in their psychology course. Students reported when they expected to complete their first draft of the course assignment, their final draft, and when they expected to submit their course project. Researchers followed up on actual completion dates 2 weeks later and found that students tended to estimate submitting their course project earlier than the actual date they submitted their course project.

While these studies are useful in understanding the way students might estimate time for their long-term goals on important tasks, they might not be as useful in understanding how students plan for short-term routine tasks. Because long-term tasks, such as an honors thesis or the rough draft of a course project, tend to be composed of smaller, everyday tasks, it is of critical importance for researchers also to consider students’ time estimation for short-term academic tasks. For example, an honors thesis is composed of many smaller tasks. While writing a thesis, a student must complete each smaller section of the paper (e.g., literature review, method, results, discussion). In addition, in these naturalistic studies, researchers tend to examine time estimation bias by focusing on the deadline by which students submit their projects. The time estimation on these smaller sections is likely important for the time estimation on the larger task.

Researchers who have focused on short-term tasks (i.e., tasks that can be completed in one relatively brief episode) have tended to adopt an experimental design. Typically, during these studies, college students are asked to estimate how long a novel task or a series of novel tasks will take in a laboratory. Then, researchers time how long the task or series of tasks takes to understand students’ time estimation accuracy. For example, in one study researchers asked students to estimate how long it would take them to sort a pile of 100 psychology journals in chronological order (Boltz and Yum, 2010). Boltz and Yum (2010) offered support for an individual’s tendency to underestimate the time these tasks would take; 72% of participants displayed an underestimation bias. In a different study, undergraduate students were asked to estimate how long it would take them to sort 500 sheets of paper into 10 equal stacks (Roy et al., 2008). The results from this study suggested that students tended to underestimate how long the sorting task would take.

Although research conducted within a laboratory offers a better understanding of time estimation bias on short-term tasks, this design is not necessarily representative of the way students typically complete academic activities or the types of academic tasks students face. When college students complete academic tasks, they must organize their schedules on their own accord. For each academic task, they can often choose when to complete the task and how much time they plan to spend on the academic task. Additionally, the tasks that they complete do not tend to be novel. Students are likely to have had some level of previous experience to inform their time estimations. For instance, most students write many papers throughout their college career. Therefore, the process of writing papers becomes a routine academic task.

Taken together, it is clear that an important next step in time estimation research is to examine students’ time estimation bias on authentic, short-term tasks. In order to address this need, our study focuses on actual academic tasks that students planned to complete in the coming week. In doing so, the present study seeks to understand whether activation of task beliefs might influence time estimation bias.

### Factors related to time estimation

Because much of the previous literature has suggested that individuals tend to demonstrate bias in their time estimations (Buehler et al., 1997; Roy et al., 2008; Boltz and Yum, 2010; Weick and Guinote, 2010), researchers have turned their attention toward understanding factors that might influence time estimation accuracy. One line of research has examined how feedback and reflections on previous tasks can prompt more accurate time estimation (Roy et al., 2005, 2008; König et al., 2015), while a second line of research has examined the impact of individual’s motivational beliefs on time estimation bias (Buehler et al., 1997).

One factor thought to influence time estimation bias for current tasks is the activation of metacognitive knowledge of time estimation. Researchers have examined how the feedback on previous instances of time estimation relates to time estimation on future tasks. Roy et al. (2008) conducted three experimental studies which manipulated whether individuals were given the number of minutes it took them to complete the task previously or the number of minutes it took another person to complete the task previously. When individuals were given information about how they performed on previous tasks or how others performed on the task, they tended to be more accurate in their estimations. These findings were consistent for tasks that were novel, lab tasks (e.g., sorting papers) and tasks that were commonplace (e.g., pumping gas). This suggests that feedback on inaccurate time estimations might prompt more accurate time estimation. A different study suggested that consideration of previous incorrect estimations of dissimilar tasks can prompt students to be more accurate on future tasks (König et al., 2015). In this study, two dissimilar tasks were completed in an hour-and-a-half long session. Undergraduate students were more accurate in their time estimations when completing the second task compared to the first. This suggests that activation of incorrect experiences of time estimation might affect students’ future time estimation accuracy. In a review of research, Roy et al. (2005) highlighted “remembering the past” as one intervention researchers have used to improve the accuracy and bias of people’s time estimation (pp. 747). In general, this manipulation has not successfully improved estimation accuracy. Roy et al. (2005) argues that this could be because people are not accurately remembering their past durations. Taken together, there is inconsistency in how reflections on prior experiences influence future estimations. This could be rooted in the focus of the reflection. Studies have tended to focus on reflecting on prior task durations, but it could be important to consider other aspects of the academic task, such as task difficulty.

A second line of research focused on understanding time estimation has investigated the impact of motivation. Research has suggested a negative relationship between motivation and time estimation accuracy (Buehler et al., 1997). Indeed, Buehler et al. (1997) conducted two studies that examined the relationship between motivation and time estimation. In the first study, individuals were asked when they planned to submit their tax return, then were later surveyed on the date they actually submitted their return. The authors considered the amount of money individuals were receiving in their tax return as a proxy for motivation to get the task completed. Results indicated that those who had larger tax refunds tended to be less accurate in predicting when they would finish the task compared to individuals who had smaller tax refunds. In study two, Buehler et al. (1997) replicated this finding with experimental methods by using money as incentives for early completion of a word generation task. Undergraduates who were offered money for early task completion tended to underestimate how long the planned tasks would take.

While these studies offer important implications for the impact of monetary incentives on individual’s time estimation accuracy, the assumption that these monetary incentives impacted individual’s motivation for the task is debateable. For example, those who have larger tax returns might also have more complex tax returns, thus making it more challenging to estimate the date they planned to submit their returns. The present study seeks to add to this work integrating the two lines of research presented above. We examined how the activation of task difficulty beliefs (i.e., prior task difficulty and anticipated task difficulty) may influence students’ time estimations. In doing so, we add to the literature by elaborating on the aspects of time estimation that might be most important for students to consider.

### A self-regulated learning view of time estimation

Although time estimation has tended to receive attention outside of the framework of time management, the process of time estimation is likely an important part of the larger system through which students control the amount of time they devote to academic tasks (Burt and Kemp, 1994; Francis-Smythe and Robertson, 1999; Wolters and Brady, 2021). In general, self-regulated learning can be defined as the active processes that students engage in as they complete academic tasks to regulate their learning (Pintrich and Zusho, 2007). Typically, self-regulated learning frameworks identify phases that occur before, during, and after students engage in an academic task (Panadero, 2017). During these phases, students can plan, monitor, control, and reflect on aspects of their learning (Zimmerman, 1990; Pintrich and Zusho, 2007). Self-regulated learning researchers describe time management as a series of processes that individuals can engage to plan, monitor, control, and reflect on their time (Wolters and Brady, 2021). Researchers have suggested that time estimation may be particularly important as students create plans for their task completion (Wolters and Brady, 2021). Of course, time estimation represents just one foundational aspect of time management. Time management involves additional subprocesses and is influenced by a range of factors. As an example, people might experience the passage of time differently (Reunanen, 2015).

Self-regulated learning is not the only framework that works to explain students’ goal attainment and academic engagement. For example, Gollwitzer (1999) suggested that people may create implementation attentions, where they create conditional statements explaining when, where, and how they will engage in particular processes. These implementation intentions are informed by goals. Prior research has suggested that the creation of implementation intentions improves the likelihood that individuals will complete their goals and are less likely to demonstrate unrealistic optimism (calculated using predicted and actual duration of the task; Koole and Van’t Spijker, 2000). Similarly, Taylor et al. (1998) argued that mental simulation can reduce the likelihood that individuals demonstrate biased task duration predictions. In addition, one study rooted in frameworks of self-regulated learning examined a five-week intervention intended to improve students’ study time calibration (Follmer et al., 2022). This study suggests that providing opportunities for students to practice estimating their time on academic tasks may lead to less biased estimations.

Central to frameworks self-regulated learning is the idea that prior task experiences influence future task experiences. Indeed, following the completion of an academic task, students may reflect on their task experience and consider effectiveness of their approaches (Zimmerman, 1990). This information is later used in future self-regulated learning cycles when students active their prior metacognitive knowledge about a particular task. Thus, students’ time estimation bias may be influenced by the activation of prior experiences with similar academic tasks. Based on frameworks of self-regulated learning, the process of time estimation might involve thoughtful consideration of a student’s (1) previous experience with the task, (2) expectations about the task, and (3) the obstacles or challenges a student anticipates when completing the task.

### Present study

The goal of this study was to expand on previous literature by investigating college students’ time estimation bias on routine, short-term tasks. First, we were interested in the time estimation bias on these academic tasks. Consistent with previous research (Buehler et al., 1997; Boltz and Yum, 2010; Buehler and Griffin, 2015), we hypothesized that students’ time estimations would be inaccurate. Specifically, we expected students to underestimate how long their academic tasks would take. Second, we investigated whether reflecting on aspects of the task would prompt students to be more accurate in their time estimation. Because research has suggested a link between previous experience with tasks and future experiences with tasks (Roy et al., 2008; König et al., 2015), we hypothesized that students who activated their task beliefs would be more accurate in their time estimation compared to students who did not. Finally, we examined the impact of students’ task related beliefs on their time estimation accuracy. Specifically, we were interested in the connection between students’ perceptions of similarity to previous tasks, difficulty of previous tasks, and difficulty of the current task to time estimation accuracy. We expected the difficulty of the previous and future task to exacerbate students’ inaccurate time estimation. This was based on previous research focused on the impact of students’ motivation on time estimation (Buehler et al., 1997). The specific research questions we examined were: (1) To what extent are college students time estimations biased?, (2) How does activation of task beliefs impact students’ time estimation bias?, and (3) How does perceived difficulty of prior tasks and anticipated difficulty of the current task relate to time estimation bias?

## Materials and methods

### Participants

Participants (*N* = 210, 58% male) were undergraduates enrolled in a learning to learn course at a large Midwestern university. All academic ranks were represented (13% first-years, 34% second-years, 26% third-years, 27% fourth-years). Students’ racial and ethnic background were based on university records; students were white (62%), Asian (11%), Black or African American (11%), two or more races (7%), or other (9%).

### Procedure

As a part of a unit focused on time management during the fifth week of the semester, all students in the course completed a two-part assignment on time estimation. However, only students who provided consent to use their data for research were included in our analyses. The assignment was accessed through the course’s learning management system. It directed students to choose an academic task that they planned to complete during the coming week and to report information about that task (i.e., type of task and goal statement). Students were specifically instructed to choose a task that they expected to complete in one sitting. After describing the task, students were randomly and surreptitiously assigned to one of two groups. Students assigned to the experimental group (*n* = 107) were required to complete four questions intended to activate their task beliefs (Table 1). Questions focused on their past experiences with the type of task they had selected, their expectations about the task, and the obstacles they expected to face while completing the task. Students in the Control group (*n* = 103) did not complete these questions. Hence, the one difference between the two groups was students’ engagement in a short, structured experience reflecting on their past experiences with similar types of tasks. Following this manipulation, all students were asked to estimate where they planned to complete the task, the day they planned to complete the task, and the time they planned to begin their task. Additionally, all students were asked to estimate how many minutes they expected the task to take. All of this information was entered into the course assignment. At the end of part 1 of the time estimation assignment, students were asked to track their actual location, day, time, and number of minutes associated with their task.

**Table 1:** Prompts completed by the experimental condition during time estimation assignment part 1.

Prompts
How similar is this task to other tasks you have completed in the past?
Were those tasks challenging for you or easy for you?
How challenging do you think this academic task will be?
What types of obstacles do you think might distract you from completing the task successfully?

Students were instructed to respond in at least one sentence for each question.

Students were instructed to complete part 2 of the assignment after they had finished their chosen task. Part 2 of the time estimation assignment was the same for all students. Part 2 of the time estimation assignment prompted students to provide information about their actual experiences completing their academic task. The first prompt asked students if they had, in fact, completed the academic task they had selected for the assignment. If students reported that they did not complete the task they were directed to open-ended questions focused on time management and time estimation and removed from our study. If students did report that they completed their academic task, they were asked to report the location, day, and time they completed their chosen task. Additionally, they were asked to report the number of minutes the task took. For the final portion of the assignment, students were asked open-ended questions focused on time management and time estimation.

### Measures

#### Task beliefs

Students’ prior perceived difficulty and anticipated difficulty with the task they had selected to use for the assignment were assessed using separate open-ended questions that were coded using steps outlined by Saldaña (2016). The first author and an undergraduate research assistant completed the coding process. First, the first author read all responses and created a coding system. Then, the research assistant was trained in the coding system. Both the first author and the research assistant independently coded a portion of responses (*n* = 27) to assess interrater reliability.

We used one of the activation questions (i.e., “Were those tasks challenging for you or easy for you?”) from the assignment to assess perceived prior difficulty. Students’ open-ended responses were categorized using codes for easy, moderately challenging, and very challenging. The interrater reliability between coders was 81%.

Anticipated difficulty was assessed using a second activation question (i.e., “How challenging do you think this academic task will be?”). Similar to previous challenge, students’ responses were coded into three categories easy, moderately challenging, and very challenging. The interrater reliability was 78%.

#### Time estimation

In part 1 of the assignment, students reported the predicted task duration (i.e., how many minutes they expected the task to take). Approximately 1 week later and after the task had been completed, they reported the actual task duration (i.e., the number of minutes the task actually took). Similar to previous studies (Boltz and Yum, 2010), a time estimation ratio was calculated as the predicted duration of task divided by actual duration of task. If a students’ ratio was less than 1, they had underestimated how long a task would take. A ratio above 1 indicated that students overestimated how long a task would take. Finally, a ratio of 1 indicated that a student’s predicted and actual time duration were the same.

## Results

### To what extent were students’ time estimations accurate?

The range, means, and standard deviations for students’ time estimation ratios, predicted task durations, and actual task durations are presented in Table 2. Students’ time estimation ratio scores ranged from .29 to 12. In terms of frequency, more students underestimated how long their selected tasks would take (47.1%) than overestimated how long their tasks would take (31.9%). A notable and surprising percentage of students (21%) reported that the actual time they devoted to the task was exactly as they had predicted. The average time estimation ratio was 1.27 (*SD* = 1.04). The time estimation ratio was not normally distributed; thus a box-cox transformation (Box and Cox, 1964) was conducted to allow for parametric analyses. The range, means, and standard deviations for the transformed time estimation ratios is presented in Table 2. All further analyses were conducted on the transformed time estimation ratio.^1^

**Table 2:** Means, standard deviations, and range of minutes spent on tasks and time estimation ratios.

Variable	Predicted task duration	Actual task duration	Time estimation
*N*	210	210	210
*M*	125.93 (2.10 h)	124.31 (2.07 h)	1.27
*SD*	86.59	99.38	1.04
Minimum	15	3	0.29
Maximum	360	480	12

Students chose the specific academic task that they planned to complete in the coming week. To ensure that there were no significant differences in time estimation bias based the particular type of task students completed, we coded tasks into three categories: reading, homework assignment, and studying for an exam. Based on results from a one-way ANOVA, there were no significant differences in students transformed time estimation ratios based on the type of task (Welch’s F (98.61, 2) = 2.29, *p* = 0.106).

### Does reflecting on tasks improve time estimation accuracy?

The transformed time estimation ratios for the experimental group were compared to those for the control group. A one-way ANOVA indicated no significant differences in time estimation ratios between the experimental and the control group (Welch’s F (198.27, 1) = 3.53, *p* = 0.06). Thus, the hypothesis that the activation of task beliefs would impact students’ time estimation bias was not supported.

### Do perceptions of the task difficulty relate to time estimation accuracy?

Analyses focused on understanding the relationship between the perceptions of task difficulty and time estimation bias included exclusively students in the experimental group (*n* = 107). A one-way ANOVA was conducted to determine whether there were differences in students’ transformed time estimation ratios based on the reported difficulty of prior tasks.

As depicted in Table 3, results indicated that students’ time estimations were significantly different based on prior difficulty (Welch’s F (65.58, 2) = 5.03, *p* = 0.009). Pairwise comparisons were performed using the Games-Howell procedure. This *post hoc* analysis indicated significant differences in transformed time estimation ratios for students whose prior academic tasks were easy (*M* = −0.01) versus challenging (*M* = 0.29; *p* = 0.007). This finding suggests that prior challenge influences students’ time estimation bias.

**Table 3:** One-way ANOVA comparing transformed time estimation ratios based on reported difficulty of previous academic task.

Variable	Easy *n* = 29	Moderately challenging *n* = 44	Challenging *n* = 34	Welch’s *F*
	Mean	Mean	Mean	
Transformed time estimation ratio	−0.01	0.08	0.29	5.03^*^

* *p* < 0.05.

Similarly, a one-way ANOVA was conducted to examine differences in students’ transformed time estimation ratios based on anticipated task difficulty (Table 4). Results indicated significant differences in transformed time estimation ratios based on students’ anticipated difficulty (Welch’s F (46.95, 2) = 3.97, *p* = 0.03). Subsequently, Games-Howell was used to perform pairwise comparisons. *Post hoc* analyses indicated significant differences between tasks that were expected to be easy (*M* = 0.03) and challenging (*M* = 0.34; *p* = 0.03). In addition, there were significant differences between tasks that were expected to be moderately challenging (*M* = 0.06) and challenging (*M* = 0.34; *p* = 0.04). This finding suggests that anticipated challenge influences students’ time estimation accuracy.

**Table 4:** One-way ANOVA comparing transformed time estimation ratios based on reported anticipated difficulty.

Variable	Easy *n* = 28	Moderately challenging *n* = 55	Challenging *n* = 22	Welch’s *F*
	Mean	Mean	Mean	
Transformed time estimation ratio	0.03	0.06	0.34	3.97^*^

* *p* < 0.05.

## Discussion

The ability to manage time effectively is an essential skill for college students (Kitsantas et al., 2008; Wolters et al., 2017). One process related to time management that could be especially important is students’ time estimation. Previous research has suggested that individuals tend to be biased in their time estimations (Buehler and Griffin, 2015). Because of this bias, researchers have worked to examine factors that might improve individual’s time estimation accuracy (Buehler et al., 1997; Roy et al., 2008; König et al., 2015). The purpose of the present study was to investigate whether the activation of task beliefs influenced students’ time estimation.

### Time estimation bias

The majority of students demonstrated biased time estimations. When comparing students who underestimated their time on academic tasks and those who overestimated their time on academic tasks, a greater number of students underestimated their time on academic tasks in the present study. This finding is in line with prior studies that have suggested that people tend to underestimate their time on tasks (Buehler and Griffin, 2015).

We hypothesized that the majority of students would underestimate the amount of time it would take to complete academic tasks, thus it was interesting that a large group of students overestimated their time on academic tasks. There are at least two potential explanations for this overestimation. First, it could be that when college students consider short-term, typical tasks they are more likely to overestimate the amount of time tasks will take rather than underestimate. Previous studies tended to find underestimation when individuals were asked to report on novel, experimental tasks (Roy et al., 2008; Boltz and Yum, 2010) or when individuals completed typical, long-term tasks (Buehler et al., 1994, 1997). In contrast to previous studies, the students in this study chose tasks with which they tended to be familiar. Even when the content of tasks was not similar to previous tasks, the types of tasks students completed were routine college tasks (e.g., reading, studying for exams, and completing homework assignments). Additionally, students chose tasks that they planned to complete in one episode, rather than tasks that would take place over multiple episodes.

A second reason why students might have overestimated the amount of time for their academic tasks could be related to the context of the study. Students completed this assignment during the time management unit of a learning to learn course (Hofer et al., 1998). Receiving direct time management instruction might have encouraged some students to be more deliberate when estimating their time on academic tasks. These students might have strategically reported predicted task durations that were longer than expected to ensure they had enough time to engage in their academic task. When comparing overestimation and underestimation, the consequences of planning too few minutes for an academic task seem to outweigh the consequences of planning too many minutes for an academic task. For example, if a student is planning to complete a lab report and allots one hour of time, if that student does not finish the lab report in the allotted time, then they might not turn in a completed lab report. In contrast, if a student allots two hours to finish their lab report and finishes the lab report in one and a half hours, then the student is able to turn in a completed lab report.

Future researchers should continue to assess the time estimation bias of college students. In particular, to clarify the reasons why students might tend to overestimate or underestimate, additional studies should use process-oriented approaches to examine the way students estimate their time. For example, think-aloud protocols could be a useful way to examine why students might overestimate or underestimate how long tasks would take. This would help researchers better understand the underlying mechanism of time estimation.

#### Influences on time estimation bias

A second major finding from this study was that students who activated their task beliefs and students who did not activate their beliefs showed similar patterns in their time estimation bias. This was surprising based on previous literature that suggested that consideration of prior experiences completing similar tasks may be a viable way to improve the accuracy of students’ time estimation (Roy et al., 2008; König et al., 2015) and literature suggesting the importance of activation of beliefs during self-regulated learning (Pintrich and Zusho, 2007).

There are at least two potential reasons why activation of beliefs about prior difficulty, anticipated obstacles, and anticipated difficulty might not have influenced time estimation in this study. First, it could be that the activation questions did not stimulate deep enough thought about students’ beliefs. In the present study, the assignment instructions asked students to respond to each question in at least one sentence. Many students who responded to the prompts did not provide one sentence, rather they provided a couple of words. For example, in response to the question “How similar is this task to other tasks you have completed in the past?” many students responded simply “similar.” When students provided one-word responses, they might not have been thinking deeply enough about their past task experiences. For instance, although students considered the task difficulty, it might have been more useful to contemplate the specific aspects of the task that they perceived as most difficult.

A second reason why the activation questions might not have prompted more accurate time estimations is due to the focus of the questions. It might be more important for students to consider different task-related beliefs (i.e., beyond prior difficulty, anticipated obstacles, and anticipated difficulty) to encourage more effective time estimation. For example, a question focused on the specific struggles students faced during previous academic tasks might have provided additional information to appropriately plan for the current academic task, thus impacting students’ time estimation accuracy. Additionally, it could be that students’ reflections prompted the recall of inaccurate memories about prior task experiences (Roy et al., 2005). Biased or inaccurate memories may have led to biased or inaccurate plans.

To continue to develop an understanding of the impact of the activation of task beliefs on students’ time estimation accuracy, future researchers could prompt deeper engagement in reflections. For instance, rather than asking students to provide a one-sentence reflection their prior difficulty, anticipated obstacles, and anticipated difficulty, researchers might ask students to respond in one paragraph or require them to address prompts for specific information that demand greater reflection and recollection of past experiences. Questions could focus on other beliefs held by students (e.g., motivational beliefs) and other perceptions of the task (e.g., task complexity).

#### The importance of perceived difficulty

A third major finding of the present study related to students’ specific beliefs about their academic tasks. Findings suggested differences in students’ time estimation ratios based on perceived prior and anticipated task difficulty. In addition to emphasizing the importance of perceived difficulty, this finding also may suggest that variation in students’ time estimation biases could be due to the specific beliefs they hold rather than whether those beliefs are explicitly activated prior to beginning an academic task. In addition, these findings suggest that difficulty could be especially important as students enact their time management processes.

In general, this seems to be in line with prior research that suggests that individual’s perceptions influence their time estimation accuracy (Buehler et al., 1997) and that task beliefs influence student’s self-regulated learning processes (Pintrich and Zusho, 2007). Of course, students’ perceptions of prior and anticipated difficulty are likely closely connected to their task experiences. As an example, difficulty may be related to students’ familiarity with a task. As prior research has suggested relationships between time estimation bias and familiarity with tasks (Boltz et al., 1998; Roy and Christenfeld, 2007), future research is needed to disentangle the task experiences and beliefs that relate to time estimation bias.

### Limitations and future directions

When considering the implications of this study, it is important to acknowledge a couple of limitations. First, all students who participated in the present study were enrolled in a learning-to-learn course, which focused on improving students’ use of self-regulated learning strategies. Future research should explore similar research questions with students who are not enrolled in a learning-to-learn course.

In addition, there are a few minor aspects of the time estimation assignment directions that might have affected the study results. First, the instructions asked students to choose a task that they planned to complete in the coming week. Although we gave students an opportunity to indicate that they did not end up completing the academic task, the majority of students might have been more likely to stick to their time estimation plan because they felt like it was part of their course assignment. This is particularly true for students who completed more flexible assignments, like reading or studying for an exam. In addition, factors outside of the scope of this study may influence time estimation and, more broadly, time management. Future studies should investigate college students’ time estimation bias outside of a learning to learn course assignment. This might alleviate additional pressure students feel to follow-through with their plan because of course assignment, making the study more ecologically valid. This may also allow researchers to explore additional factors that influence time management.

In light of the limitations associated with this study, future research should continue to explore time estimation accuracy in ecologically valid contexts with different groups of students. Exploring the factors that impact students’ time estimation continues to be an important goal because of the potential connection to students’ self-regulated learning and time management. Viewing time estimation through a lens of self-regulated learning offers specific factors that could be investigated in relation to time estimation accuracy, like students’ motivational beliefs, other time management strategies, or the tendency to procrastinate. Better understanding the relationship between time estimation and other aspects of self-regulated learning would provide insights into the role of time estimation.

### Conclusion

Time management has been viewed as an important process for college students’ academic success (Zimmerman et al., 1994; Claessens et al., 2007; Kitsantas et al., 2008; Wolters et al., 2017). Time estimation is one potentially important aspect of time management. This study contributes to previous work focused on time management and time estimation by investigating students’ time estimation accuracy for typical short-term academic tasks. Findings indicate that students tend to be biased in their time estimations, and that reflecting on previous tasks does not influence students’ time estimation bias. In addition, findings highlight that both prior task difficulty and anticipated difficulty may influence students’ time estimation bias.

## Data availability statement

The datasets presented in this article are not readily available because we did not receive IRB approval to share the data sets. Requests to access the datasets should be directed to abrady@georgiasouthern.edu.

## Ethics statement

The studies involving human participants were reviewed and approved by The Ohio State University - IRB. The patients/participants provided their written informed consent to participate in this study.

## Author contributions

AB collected and analyzed the data and completed the majority of the writing. CW helped with the study design, data analysis approach, and revised writing. SY helped with the study design and revised the writing. All authors contributed to the article and approved the submitted version.

## Conflict of interest

The authors declare that the research was conducted in the absence of any commercial or financial relationships that could be construed as a potential conflict of interest.

## Publisher’s note

All claims expressed in this article are solely those of the authors and do not necessarily represent those of their affiliated organizations, or those of the publisher, the editors and the reviewers. Any product that may be evaluated in this article, or claim that may be made by its manufacturer, is not guaranteed or endorsed by the publisher.

## Footnotes

^1^All analyses were also conducted using Kruskal–Wallis *H*-tests, the nonparametric version of the ANOVA. The Kruskal–Wallis *H*-tests produced similar results.
