# Corrigendum: Self-regulation of time: The importance of time estimation accuracy

**DOI:** 10.3389/fpsyg.2022.1094993

**Published:** 2023-01-05

**Authors:** Anna C. Brady, Christopher A. Wolters, Shirley L. Yu

**Affiliations:** ^1^Department of Curriculum, Foundations, and Reading, College of Education, Georgia Southern University, Statesboro, GA, United States; ^2^Dennis Learning Center, Department of Educational Studies, College of Education and Human Ecology, The Ohio State University, Columbus, OH, United States; ^3^Department of Educational Studies, College of Education and Human Ecology, The Ohio State University, Columbus, OH, United States

**Keywords:** self-regulated learning, time management, time estimation, planning fallacy, college students

In the published article, there was an error in Shirley L. Yu's affiliation. Instead of “Dennis Learning Center, Department of Educational Studies, College of Education and Human Ecology, The Ohio State University, Columbus, OH, United States,” it should be “Department of Educational Studies, College of Education and Human Ecology, The Ohio State University, Columbus, OH, United States.”

The authors apologize for this error and state that this does not change the scientific conclusions of the article in any way. The original article has been updated.

